# Temporal Unfolding of Micro-valences in Facial Expression Evoked by Visual, Auditory, and Olfactory Stimuli

**DOI:** 10.1007/s42761-020-00020-y

**Published:** 2020-11-13

**Authors:** Kornelia Gentsch, Ursula Beermann, Lingdan Wu, Stéphanie Trznadel, Klaus R. Scherer

**Affiliations:** 1 Swiss Center for Affective Sciences (CISA), University of Geneva, Campus Biotech, 9, Chemin des Mines, CH-1202 Geneva, Switzerland; 2 Erfurt, Germany; 3 Department of Psychology, UMIT-Private University for Health Sciences, Medical Informatics and Technology, Hall in Tirol, Austria; 4 Lyon, France; 5 Wyss Center for Bio- and Neuroengineering, Geneva, Switzerland

**Keywords:** Emotion process, Valence appraisal, Micro-valences, Intrinsic pleasantness, Goal conduciveness, Facial electromyography

## Abstract

Appraisal theories suggest that valence appraisal should be differentiated into micro-valences, such as intrinsic pleasantness and goal-/need-related appraisals. In contrast to a macro-valence approach, this dissociation explains, among other things, the emergence of mixed or blended emotions. Here, we extend earlier research that showed that these valence types can be empirically dissociated. We examine the timing and the response patterns of these two micro-valences via measuring facial muscle activity changes (electromyography, EMG) over the brow and the cheek regions. In addition, we explore the effects of the sensory stimulus modality (vision, audition, and olfaction) on these patterns. The two micro-valences were manipulated in a social judgment task: first, intrinsic un/pleasantness (IP) was manipulated by exposing participants to appropriate stimuli presented in different sensory domains followed by a goal conduciveness/obstruction (GC) manipulation consisting of feedback on participants’ judgments that were congruent or incongruent with their task-related goal. The results show significantly different EMG responses and timing patterns for both types of micro-valence, confirming the prediction that they are independent, consecutive parts of the appraisal process. Moreover, the lack of interaction effects with the sensory stimulus modality suggests high generalizability of the underlying appraisal mechanisms across different perception channels.

**Electronic supplementary material:**

The online version of this article (10.1007/s42761-020-00020-y) contains supplementary material, which is available to authorized users.

## Introduction

The concept of valence is generally considered as a central feature of emotion experience (and “probably the most promising criterion for demarcating emotion from cognition”; Charland, 2005, p. 82). Emotion researchers mostly use valence to describe the positive or negative quality of emotion or its components such as subjective feelings, expression, and behavioral responses (Brosch & Moors, 2009). In a large-scale intercultural study of the semantic structure of emotion terms in 23 different languages, Fontaine, Scherer and Soriano (2013) confirmed that valence is by far the most powerful of the four basic dimensions differentiating them. However, given that many languages have several hundred emotion terms, it would be highly unsatisfactory to reduce this variety to a distinction between positive and negative emotions. Emotion theorists trying to understand the nature and the process of emotional experience have taken a more discriminating approach. In particular, several appraisal theories distinguish different types of valence appraisal, such as the appraisal of hedonic pleasantness a person associates with particular objects or situations, as compared to appraisal based on motivational criteria, such as the degree of compatibility of an event to a person’s needs, goals, and values (Ellsworth & Scherer, 2003; Scherer, 1984a, 1984b; Smith, 1989). Shuman, Sander and Scherer (2013) have proposed distinguishing these micro-valences from a one-dimensional macro-valence.

This distinction plays an important role in the Component Process Model of emotion (CPM; Scherer, 1984a, 2001, 2009) which differentiates between appraisals of intrinsic pleasantness (IP, appraising objects or situations with respect to the sensual or hedonic experience they elicit) and of goal conduciveness (GC, appraising events and their consequences with respect to whether they help or hinder goal attainment). The model further suggests that appraisal outcomes about an event or an object drive the changes in the emotion response components (i.e., action tendencies, physiological reactions, facial and vocal expression, as well as subjective feeling). The emergent appraisal-specific response patterning can then be categorized and eventually labeled with a specific emotion term (see Scherer, 2001, 2009, for further details of the theory). The CPM makes specific, operationalized predictions about the appraisal-driven component patterning. A central part of the process-oriented CPM is the assumption of a sequential, recursive appraisal process that constantly updates the appraisals of the following checks or criteria: novelty, intrinsic pleasantness, goal conduciveness, causal origin, coping potential, and normative significance (for more detail and predicted appraisal patterns for discrete emotions, see Table 1 in Scherer, 2009). The most recent summary of the predictions for facial expression has been published by Scherer et al. (2017; see Table 19.1) which partially drive the hypotheses examined in this article.

**Table 1:** Component patterning theory predictions for facial muscle movements following intrinsic un/pleasantness and goal conduciveness/obstructiveness appraisal outcomes

Intrinsic pleasantness appraisal	
Pleasant	Unpleasant
Brow region	
–	Brow lowering (corrugator supercilii), lids tighten (orbicularis oculi)
Cheek region	
Lip corners pulled upwards (zygomaticus major), lips part (depressor labii)	Nose wrinkling (levator labii superioris alaeque nasi), upper lip raising (levator labii superiori), lip corner depression (triangularis), chin raise (mentalis), lips tightened and pressed (orbicularis oris)
Goal conduciveness appraisal	
Conducive	Obstructive
Lower activity of facial muscles	Higher activity of facial muscles
Brow region	
–	Brow lowering (corrugator supercilii), lids tighten (orbicularis oculi),
Cheek region	
Lip corners pulled upwards (zygomaticus major), lips part (depressor labii)	–
-	Upper lip raising (levator labii superiori), lips tightened, pressed (orbicularis oris), chin raising (mentalis)

According to this sequence assumption, the evaluation of the un/pleasantness of an event is performed prior to the evaluation of its facilitation or obstruction of goals. This is important in that it justifies the distinction between the two micro-valences and their respective functions: The IP check alerts the organism to something pleasant, desirable, and to be approached vs. something unpleasant, undesirable, to be avoided. The subsequent GC check informs the organisms on how conducive or obstructive for reaching one’s goals the respective object or situation is likely to be, preparing the appraisal of causality and coping ability, and thus preparing the optimal action tendencies.

The dual nature of valence appraisal may thus be part of the explanation for the existence and the frequent occurrence of mixed or blended emotions (see Larsen & McGraw, 2011; Miyamoto, Uchida & Ellsworth, 2010; Scherer & Meuleman, 2013). Furthermore, the distinction is central for a dynamic, process-oriented approach to emotion, especially for appraisal theories suggesting a sequence of appraisal with cumulative effects and a recursiveness of these appraisal-driven responses. This is particularly relevant for the prediction and measurement of the continuous changes in the response components which are assumed to be affected by different appraisal check results. Further justification for the need to distinguish these micro-valences is provided in Shuman et al. (2013).

To date, guided by the theoretical framework of appraisal theory and specifically of the CPM, several studies have examined the dissociation of the two types of micro-valences using high temporal resolution response measures. Generally, GC was experimentally manipulated by providing feedback (visually or orally) about outcomes that were congruent or incongruent with the person’s goals, whereas IP was manipulated by exposing the person to appropriate stimuli in different sensory domains. Some studies have used electroencephalography (EEG) (cf. Gentsch, Grandjean & Scherer, 2013; Grandjean & Scherer, 2008) to monitor the temporal unfolding of a series of appraisal checks with high temporal precision. Studies using facial electromyography (EMG) have generally focused on the distinction between IP and GC using visual (Aue, Flykt & Scherer, 2007; Aue & Scherer, 2008, 2011; Lanctôt & Hess, 2007), auditory (van Reekum et al., 2004), or olfactory stimuli (Delplanque et al., 2009). The results of these studies suggest two major findings: (1) appraisal results of IP and GC produce similar but subtly different facial response signatures and (2) these effects unfold sequentially over time in the order predicted by the CPM: IP before GC.

These consistent results suggest that the sequential effects and the respective response patterning in the dynamic unfolding of facial muscle activity changes seem to be largely independent of specific manipulations of the respective appraisal in an experimental task (e.g., the operationalization of IP and GC) and of the sensory domain in which the appraisals were manipulated, suggesting a high degree of generalizability of the findings. However, the timing of the respective response patterns after stimulus onset varies somewhat over studies. There are multiple explanations for these variations: the nature of the manipulations and the stimuli in the various sensory domains, characteristics of the participants, cultural factors, to name but some of the major factors. Therefore, further work is needed to systematically investigate both the patterning and the timing of the predicted response dynamics. In this article, we report data from a large-scale study with several controlled manipulations to determine the effects of the dynamic unfolding of the appraisal signatures of IP and GC on facial EMG. Furthermore, we investigate whether their signatures differ as a function of the sensory modality (vision, audition, olfaction) in which the stimuli were presented. In sum, this work pursues the following three aims: (1) to replicate earlier results in studies with different aims and designs showing that IP and GC effects can be clearly distinguished in a study using a homogeneous design focusing only on IP and GC differences, (2) to explore the differences in response patterning in the facial musculature as well as the respective timing both of which varied somewhat in earlier work, and (3) to examine potential interaction effects with the sensory modality of stimulus presentation which might endanger generalizability of the results.

## The Present Study

This article reports results of facial EMG responses collected as part of a larger study that included other classes of dependent variables (EEG, autonomous nervous system measures, and voice analyses) as well as an exploratory examination of potential regulation influences by the presence of another person. As it would be impossible to report this massive data set in a single article, several reports on different response measures are currently in preparation.

Past research in this area has often put the emphasis on the measurement of significant increases in activation at specific time points as a result of a particular appraisal manipulation. In some cases, combinations of different appraisal checks have been manipulated (e.g., Aue & Scherer, 2011). Importantly, in this study, we separately compare pleasant vs. unpleasant and conducive vs. obstructive appraisal outcomes, using a single experimental design with the same group of participants. Overall, we predicted that appraisal results of IP, as compared to GC, would show (a) an earlier onset (based on the sequence hypothesis of the CPM) and (b) a subtly different response pattern for facial muscle responses (based on the componential patterning predictions of the CPM). These componential patterning predictions on facial muscle movements are summarized in Table 1. In addition, we also considered the findings of earlier studies on these issues and general work of facial expression in drawing up specific hypotheses.

### Hypotheses

#### Onset and Timing Predictions

The fundamental prediction on onset and timing, based on the CPM sequence hypothesis, is that the onset of effects generated by the IP check will always occur before the onset of the effects generated by the GC check. The CPM predictions further concern the facial muscle movements involved in each appraisal-driven response. While the sequence prediction has been largely confirmed in earlier work using facial EMG measurement, the observed timing has varied somewhat over different studies (Delplanque et al., 2009; Gentsch, Grandjean & Scherer, 2015a; Lanctôt & Hess, 2007).

More precise timing of appraisal checks can be obtained by using EEG measures. Gentsch, Grandjean and Scherer (2015b, Fig. 10) summarize several EEG studies on the issue of appraisal sequence and timing. These results can be used to extrapolate assumptions about the timing of corresponding EMG responses by assuming a delay of 300–400 ms between the onset of the respective EEG pattern and the corresponding EMG signal (as suggested by data reported in Mavratzakisa, Herbert & Wall, 2016). Again, timing varies somewhat over the different studies. This variation is to be expected because of the respective level of processing (see Leventhal & Scherer, 1987) and because of the complexity of the appraisal operations which vary due to differences in the type and the sensory modality of the respective experimental stimuli. Based on these results, we estimated a time window between 400 and 1600 ms during which the respective responses can be reasonably expected to reveal sequential effects of IP and GC. We also anticipated the IP response to have a shorter duration, as it is generally elicited immediately after the perception of an object, whereas GC may require a lengthier cognitive process to evaluate whether the perceived object is conducive or obstructive to a current (task-related) goal. As we are not making concrete predictions on latency and timing differences, the respective results will be discussed in an exploratory fashion.

#### Patterning Predictions (Based on Table 1)

### IP Appraisal

#### Pleasant Outcome

While no changes are expected in the brow region, we expect a strong increase in the cheek region (lip corners pulled upwards, lips part).

#### Unpleasant Outcome

We expect an increase in activation for the brow region (brow lowering, lids tightening) as well as for the cheek region (nose wrinkling, upper lip raising, lip corner depression, lips tightening).

### GC Appraisal

#### Conducive Outcome

We expect a generally lower level of activity of the facial muscles as there is little need for action preparation. However, there might be a small amount of activation in the cheek region (lip corners pulled upwards). We do not expect changes in the brow region.

#### Obstructive Outcome

We expect a generally higher level of muscle activity changes due to the need of action preparation. Specifically, we expect an increase of activity in the brow region (brow lowering, lids tightening) and in the cheek region (upper lip raising, lips tightened).

In these hypotheses, we specify the individual facial muscles expected to be activated by IP and GC appraisal results (as listed in the CPM predictions, see Table 1). However, these predictions cannot be directly tested as current facial EMG technology does not allow to measure the innervation of individual muscles in the face. The major measurement domains are relatively undifferentiated, i.e., brow and cheek regions. The brow region is mostly associated with the innervation of the corrugator supercilii (frown), but other facial muscles, for example around the eyes, may contribute to higher activation in this area. The cheek region is generally expected to be indicative of the zygomaticus major muscle (smiling) but given the density of different facial muscles in the cheek region, increased activation may well be due to other muscles around the mouth (see also “Material and Method”)*.*

#### Sensory Modality Effects

In the absence of theoretical predictions, we expected the IP and GC response differences to be largely similar across the sensory modalities in which the eliciting stimuli were perceived (given the fact that similar results were found in studies using stimuli presented in different sensory modalities; e.g., Aue & Scherer, 2011; Delplanque et al., 2009; Grandjean & Scherer, 2008; Lanctôt & Hess, 2007; van Reekum et al., 2004). However, we feel that it is important to systematically examine potential modality differences within the same design using a single group of participants to obtain more reliable indications on both the patterning and the timing of the EMG responses. As we did not make specific predictions, the resulting data are explored orienting on the theoretical predictions of the CPM.

## Material and Method

### Participants

Forty-eight right-handed healthy students (23 males; mean age = 23 years, SD = 4.30) participated in the experiment for payment. They were recruited through advertisements in the university and mailing lists. All had normal or corrected-to-normal vision, normal hearing and smelling capacities. The study was approved by the Geneva Psychology Research Ethical Committee. Written consent was obtained from all participants. Individuals suffering from hearing problems, a cold, a history of psychiatric or neurological disorders, or a head injury were excluded from participation.

### Experimental Design

In an earlier study by our group (Aue & Scherer, 2011), IP, GC, and anticipation had been tested in a 2 × 2 × 2 within design, measuring the EMG responses for all eight combinations. The analysis showed many significant interactions, which made it difficult to evaluate the main effects of the valence appraisals. For this reason, in this study, we used separate within-designs for IP and GC. This seemed to be even more indicated because the major aim of the study was to investigate the effect of three presentation modalities on facial muscle activity changes. Moreover, using separate within-designs in conducting the study was realistic (in terms of economic and practical concerns) given the limited number of participants that could be recruited for a lengthy study with two sessions over three months. The modality of the stimuli to be presented requires meeting specific criteria for un/pleasantness that differ between modalities, e.g., symmetry for visual stimuli, spectral sound characteristics for auditory stimuli, and specific fragrances for olfactory stimuli. To reduce these modality-specific aspects and provide a comparable context for the stimulus presentation, we operationalized this appraisal variable in terms of the degree to which a person’s face is pleasant to look at, a person’s voice pleasant to listen to, and an odor, presumably preferred by a person, that is pleasant to smell. GC was experimentally manipulated, similar to our previous studies (Gentsch et al., 2015a, 2015b), by producing a success experience feedback (being told to have made a correct judgment and receiving a small bonus) versus a failure experience feedback (being told to have made an incorrect judgment and not receiving a bonus).

As IP and GC require quite different types of manipulations, object-oriented vs. outcome-oriented, they cannot be directly compared in an overall factorial design that allows direct examination of the significance of the differences between micro-valence types. Therefore, the differences regarding the onset times and the magnitude of differences between pleasantness vs. unpleasantness, and goal conduciveness vs. goal obstructiveness effects are evaluated on a descriptive level, using the theoretical predictions as guidelines.

### Selection of Modality-Specific Stimuli

We used existing corpora of faces, voices, and odors and ran two pilot studies to select the appropriate stimuli (for operationalizing IP) and the presentation procedure, separately for (1) visual and auditory stimuli and (2) olfactory stimuli.

#### IP Stimuli

##### Visual Stimuli

We chose human faces since they have the advantage that basic features (eyes, nose, mouth, etc.) are the same across all visual stimuli. In addition, they are equally familiar to all participants. Thus, candidate stimuli for pilot study 1 (facial portraits of female and male persons between 18 and 40 years old) were retrieved from the Face Research database (DeBruine & Jones, retrieved 2015, Face research lab, University of Glasgow Institute of Neuroscience and Psychology), the Dallas Face database (Minear & Park, 2004) and the Radboud Faces database (Langner et al., 2010). To increase the number of pleasant faces (in total, we had 236 portraits of different faces) and to amplify the difference between pleasant and unpleasant faces, we used the beautification tool in ArcSoft PhotoStudio6 (ArcSoft, Inc., version number 6, installed 2015). Digital beautification is considered to increase the pleasantness of the face. It comprises skin smoothing (reducing blemishes, wrinkles, spots, freckles), eye brightening, and face color adjustment. Additionally, we morphed some photos by using the online feature “Make an average” (DeBruine & Jones, feature used 2015, Face research lab, University of Glasgow, Institute of Neuroscience and Psychology, http://www.faceresearch.org,) in order to increase the symmetry between the facial features (cf. DeBruine, 2004). The opposite procedure, increasing the unpleasantness, was applied to the other half of the faces by using MakeUpInstrument, n.d. (Version 6.8, www.makeupinstrument.com). In particular, the symmetry of a face was reduced and features that make a face pleasant to look at were reduced (e.g., eyes and lips made smaller, ears made bigger, full hair reduced). Moreover, for all chosen stimuli, low level-features such as luminance, contrast, and spatial frequency were controlled and adjusted. Moreover, their size (300 × 400 pixels) was equalized and they were presented on a light gray background. The portraits were presented for 1.7 s.

##### Auditory Stimuli

We chose voices to correspond maximally to the face stimuli as part of human individuality. In addition, like faces, they are equally familiar to all participants. There is evidence that voices vary substantially in pleasantness. Unpleasant voices are often raspy, grating, husky, or shrill. In addition, there are distinctive voice parameters that are strongly related to the perceived pleasantness of a voice, such as Harmonic-to-noise ratio (HNR, *r* = .66**), jitter (*r* = − .65**), or shimmer (*r* = − .70**; see Beermann, Gentsch, Wu, Trznadel & Scherer, 2015). The auditory stimuli (258 voice samples from speakers between 20 and 45 years old) were obtained from the Saarbruecken voice database which contains voices of healthy persons and persons with massive voice pathologies (Pützer & Barry, n.d.). The stimuli consisted of female and male speakers articulating three vowels in a fixed sequence (/a/, /i/, /u/). The loudness of the voice samples was equalized to 70 dB. Standard length was obtained by presenting each vowel for 500 ms with an interstimulus interval of 100 ms, yielding a total length of 1.7 s.

##### Pilot Study 1: Procedure

In total, 53 participants (37 females, 16 males; between 18 and 43 years old, *M* = 24.30, SD = 4.79) were recruited via e-mail lists and advertisements in exchange for monetary compensation (15 CHF). The study was implemented in an online survey. Participants rated the degree of pleasantness and unpleasantness (on a nine-point scale from − 4 (very unpleasant) to + 4 (very pleasant)) experienced when watching the portraits or listening to the voices.

To ensure high quality and comparable conditions for stimulus presentation, participants were asked to watch the portraits on a computer screen and not on their smartphone or tablet. Furthermore, they were instructed to wear headphones and to make sure that the sound system of their computer is turned on and that they can hear the sound at an appropriate volume. Even though the quality of headphones can vary, the quality variation is considerably lower than what is to be expected with different loudspeakers, which vary from monitor loudspeakers to high-end audiophile loudspeakers. Thus, we ensured an overall better and more homogenous sound quality, and we reduced the effects of disturbing noises from the participants’ environment by instructing them to use headphones. To obtain a measure of sound quality, we asked participants to rate on a five-point scale from − 2 (not at all) to + 2 (very well), how well they were able to hear the sound prior to their ratings of the stimuli. The average rating of sound quality was *M* = 1.98 (SD = 0.46), with a minimum of 0 (ok). This result suggests a comparable level of high sound quality across participants.

For both visual and auditory stimuli, ratings were obtained in three groups, respectively, with the number of raters ranging from 15 to 19. Cronbach’s interrater reliability coefficients were consistently higher than .90, in most cases, .95. Based on participants’ ratings, 52 visual stimuli (portraits, 26 women, 26 men) and 52 auditory stimuli (voice samples, 26 women, 26 men) were selected for the main study. We selected the same number of female and male portraits and voice samples for the pleasant and the unpleasant stimuli to control for possible gender effects.

##### Pilot Study 2: Olfactory Stimuli

A set of 25 odor samples, likely to be perceived as pleasant or unpleasant, were provided by Firmenich SA, a major fragrance and flavor company in Geneva. The selection was based on empirically retrieved expert opinions of the providing company and on previous research studies carried out by Delplanque and colleagues (e.g., 2009).

Due to the technical requirement for olfactory stimulus presentation, we recruited 13 PhD and postdoctoral students (7 females, 6 males; between 27 and 43 years old, *M* = 32.62, SD = 4.44) participating on a voluntary basis. In a laboratory of the Swiss Center for Affective Sciences, the participants rated the pleasantness on a nine-point scale from − 4 (extremely unpleasant) to 4 (extremely pleasant), the intensity of the odors on a scale from 0 (not intense at all) to 8 (extremely intense), and the familiarity on a scale from 0 (not familiar at all) to 8 (extremely familiar).

Cronbach’s interrater reliability coefficient reached .97 for the pleasantness ratings. Based on these ratings, 18 odor stimuli (nine pleasant and nine unpleasant odors of comparable intensity and familiarity) were selected for the main study.

#### GC Stimuli

Participants were asked to judge whether the respective stimulus person was likely to be an extraverted or an introverted individual. They were asked to announce their judgment by saying the word “extra” if they judged the person to be extraverted, or “intra” if they judged the person to be introverted. GC was systematically manipulated by giving feedback to the participants on whether their judgment on the personality based on the face, the voice, or the odor that was smelled (requested for each stimulus) was correct or incorrect. For each judgment, the participants received the predetermined feedback alternative. In the case of the “correct” feedback, participants received a small amount of money as a bonus (in total, each participant received a bonus of 20 CHF).

We felt that the presentation modality of the feedback should be aligned as much as possible to the respective stimulus modality and timing to avoid noise due to interference.

##### Visual Stimulus Modality/Visual Feedback

The feedback stimuli consisted of a single word: either “correct” (“vrai” in French) or “incorrect” (“faux” in French) presented in the center of the screen. The low-level features of the visual feedback stimuli such as luminance, contrast, and spatial frequency screen were controlled and adjusted to the parameters of the portraits. In addition, the presentation time of the feedback was aligned to the IP stimuli presentation. Feedback was presented for 1.7 s.

##### Auditory Stimulus Modality/Auditory Feedback

Here, the feedback stimuli consisted of recorded vocal utterances: “correct” (“vrai” in French) or “incorrect” (“faux” in French). The loudness was set to 70 dB and the length was 1.7 s, as was the case for the IP stimuli.

##### Olfactory Condition/Audiovisual Feedback

Because feedback in the form of specific odors is not feasible, we presented audiovisual feedback stimuli (“correct” or “incorrect”; shown on the screen and simultaneously presented as a vocal utterance) to create a third feedback condition distinct from the two others.

### Procedure

To assess the exclusion criteria (see above), participants answered a background questionnaire online before they came to the laboratory. In addition to the relevant questions concerning their health status for the potential exclusion, participants were asked to complete a set of disposition assessment scales.^1^ These data were not used in the current analysis.

Participants were told that they were taking part in an experiment on social perception in which central and peripheral bodily reactions are recorded (not reported here). The experimental task consisted of indicating whether the person to be judged, whose face, voice, or preferred odor were experimentally presented, was likely to be more introverted or more extraverted. They all participated in two laboratory sessions one week apart. When they arrived in the laboratory for their first session, participants signed an informed consent form and answered an additional health status questionnaire. Female participants were asked whether they are on hormonal contraception and at which stage of their cycle (beginning, middle, end) they were on the day of the recordings. Electrodes were then placed, and participants were seated in front of the computer, followed by a 5-min relaxation period. Finally, participants were familiarized with the experimental task by performing two trials in each modality condition.

**Fig. 1:**
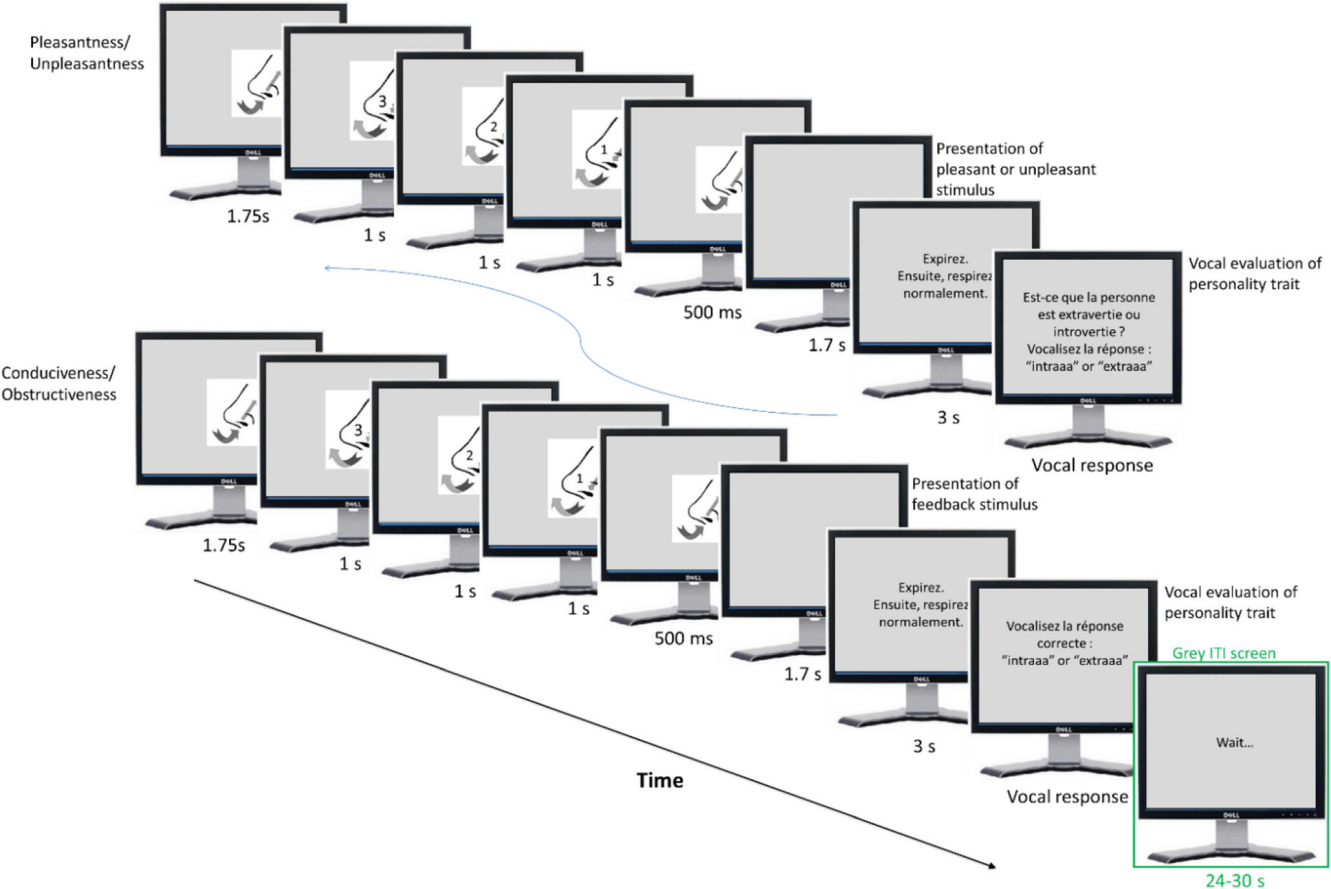
Trial structure of the experimental task. Note: Upper row shows the screens of the intrinsic pleasantness part of the trial. At the beginning of each trial, the participant’s breathing cycle was synchronized: Participants were instructed to breathe in and then breathe out by counting 3-2-1. This allowed them to breathe in with each stimulus onset. After each stimulus presentation, participants were asked whether the person is extraverted or introverted. Participants responded by saying either “extra” or “intra.” The goal conduciveness part was then manipulated starting with synchronizing the breath. This allowed participants to breathe in at the same time as the stimulus was presented. Next, they were prompted to vocalize the correct response by saying either “extra” or “intra.” After a short break, a new trial started. The experimental stimuli for each modality were presented in separate blocks

The experimental task consisted of being exposed, in separate blocks of trials, (a) to portraits presented on a computer screen (17″, resolution 1280 × 1024, sitting at a distance of 80 cm resulting in stimulus eccentricity of 5.68° × 7.57° visual angle), (b) to vocal samples expressing vowels presented via loudspeaker, and (c) to odors presented by a professional odor delivery device (see Delplanque et al., 2009). Each experimental trial (see Fig. 1: upper part, for an example) started with a centrally presented fixation cross (jitter 1.5–2 s), which participants were instructed to look at and to start controlling their breath. This was necessary to make sure that all participants always started to breath in as soon as an odor was delivered by the odor delivery device. The presentation of odors follows a specific protocol to minimize the intra- and inter-participant breathing pattern variability, a procedure that is described in earlier work (e.g., Jung et al., 2006). Following this specification, we applied the same breathing instructions to all experimental conditions to avoid an additional noise factor. Thus, participants started to breathe in with the presentation of the fixation cross and to breathe out with the visually presented countdown (3-2-1); they then breathed in again with the onset of each stimulus presentation (cf. Royet et al., 2000). The respiratory cycle prior to each stimulus was about 4 to 5 s. Regarding our dependent measures, we deemed it necessary to synchronize the respiratory cycle for all experimental blocks (vision, audition, olfaction) to minimize noise derived from different breathing cycles.

Next, the IP stimulus (portrait, voice, or odor) was presented (1.7 s) and the participant indicated by vocalizing “intra” or “extra” whether this portrait or voice stemmed from an introverted or extraverted person, or whether the odor was preferred by someone who is introverted or extraverted. This response format was chosen because of the credibility of the presumed link between face, voice, and odor and the personality of the senders, as well as to obtain a sustained /aa/ sound for off-line acoustic analyses (not reported here). Right after the participant’s response, the systematically manipulated GC feedback (“correct” or “incorrect”) was presented (1.7 s). The participant then had to pronounce the presumed “correct” response: i.e., repeat the initial response if the feedback had been “correct” or change the initial response if it had been “incorrect” (again by vocalizing either “intra” or “extra”). A new trial started immediately thereafter. Each stimulus modality was presented in separate blocks (the visual and auditory blocks each consisted of 52 stimuli, the olfactory block of 18 stimuli). In total, 122 trials were presented. Participants were allowed a short break after each block. Participants received 100 CHF for their participation and all received a bonus of 20 CHF, in total, every participant was paid 120 CHF.

There were two laboratory sessions with one week in between. The sessions were identical in terms of experimental task, stimuli, and timing. However, in one of the two sessions, a Social Presence condition was added (presence of a confederate in the lab room). As the data for the social presence condition are to be reported in a separate article, only the data from the alone sessions are reported here.

As a manipulation check, at the end of the experiment, and only after the second session, participants rated each stimulus concerning the felt pleasantness (nine-point scale from 1 [very unpleasant] to 9 [very pleasant]). The pleasant stimuli were perceived as significantly more pleasant than the unpleasant stimuli (*t*(36) = 11.53, *p* < .001, Cohen’s *d* = 1.90 for visual stimuli, *t*(36) = 10.91, *p* < .001, Cohen’s *d* = 1.79, for auditory stimuli, and *t*(36) = 11.77, *p* < .001, Cohen’s *d* = 1.94 for olfactory stimuli; for more detail see Table S1 in SOM). This result indicates that the IP manipulation was as intended and successful.

### Data Acquisition

The experimental task and behavioral data acquisition were administered by using E-Prime 2.0 (Psychology Software Tools, Inc., Pittsburgh, PA). Facial EMG was applied to assess the predicted facial muscle innervations produced by the manipulated appraisals. The surface electrodes were placed over the brow region (mainly targeting the corrugator supercilii muscle) and the cheek region (mainly targeting the zygomaticus major muscle, according to the established guidelines; Fridlund & Cacioppo, 1986). Signals were recorded (bandwidth 0.1–417 Hz, sampling rate: 2048 Hz) with a BIOSEMI Active-Two amplifier system (BioSemi Biomedical Instrumentation, Amsterdam, the Netherlands).^2^

As mentioned in the “Introduction,” surface EMG on the face does not allow recording of the activity of individual muscles, but only the overall activity over a circumscribed region, in this case the brow and cheek regions. Thus, the recorded signals cannot be unequivocally attributed to the muscle movements that are theoretically predicted. Hess (2009, p. 83) has pointed out that cross-talk between muscles can produce unexpected results. For instance, the masseter muscle, often active in anger expressions (clenching of the teeth) is a much stronger muscle than the zygomaticus major muscle, which is involved in smiling. As the electrode placements for these two muscles are close to each other, cross-talk between these two muscles can lead to absolute higher values of zygomaticus major activity being observed during biting related responses (e.g., occurring with goal obstructiveness or high power appraisals) than during smiling (elicited by goal conduciveness) (see Gentsch et al., 2015a, 2015b). Moreover, Hess et al. (2017) examined the internal consistency of EMG measures for affective reactions on facial muscles to pictorial stimuli and reported that measures of the brow region had higher internal consistencies compared to the cheek region. Importantly, in the absence of a strong zygomaticus innervation, it is likely that cheek region activation can also indicate the innervation of other neighboring muscles, such as the levator labii superioris alaeque nasi, the levator labii superiori, and orbicularis oris, all of which are predicted by the CPM for unpleasantness and obstruction (see Table 1).

### Data Analysis

Preprocessing of the EMG data (Brain Vision Analyzer software, Brain Products, Gilching, Germany) followed the standard procedure (Fridlund & Cacioppo, 1986). First, bipolar montages were calculated for each pair of electrodes of each facial muscle region (brow and cheek region) by subtracting the recorded activity of one electrode from the activity of the neighboring electrode. Next, the continuous waveforms of the EMG data were bandpass filtered (20–400 Hz, 12 db/octave), full-wave rectified, low-pass filtered (40 Hz, 12 db/octave), and cut into segments (including 500-ms baseline and 1.6-s post-stimulus intervals) for each experimental condition. The EMG data were then downsampled to 512 Hz and exported to a commercial software package (MATLAB R2012a). Separately, for each facial muscle region, artifacts and outliers (i.e., activation deviating more than two standard deviations from the mean baseline activation of a given participant) were eliminated (in total, 3.87% of all trials). To examine the temporal profiles of facial EMG for 1.6 s after each stimulus, we calculated mean amplitude values for the subsequent 100-ms time intervals as a percentage change of the mean amplitude value of the baseline.

### Statistical Analysis

The EMG data, separately for the brow and the cheek regions, were submitted to two repeated measures multivariate analysis of variance (MANOVA): (a) to a 2 (IP: pleasant vs. unpleasant) × 3 (modality: vision vs. audition vs. olfaction) × 13 (time: 100-ms time intervals from 400 to 1600 ms) and (b) to a 2 (GC: correct vs. incorrect feedback) × 3 (modality: vision vs. audition vs. olfaction) × 13 (time: 100-ms time intervals from 400 to 1600 ms). The within-subject variables were IP or GC, and modality. Time was introduced as a multiple dependent variable represented by the repeated measures (see Delplanque et al., 2009; Gentsch et al., 2015a, 2015b). In addition, for each 100-ms time interval, the variance difference among the experimental conditions was tested by using univariate within-subjects tests (planned comparisons).

**Fig. 2:**
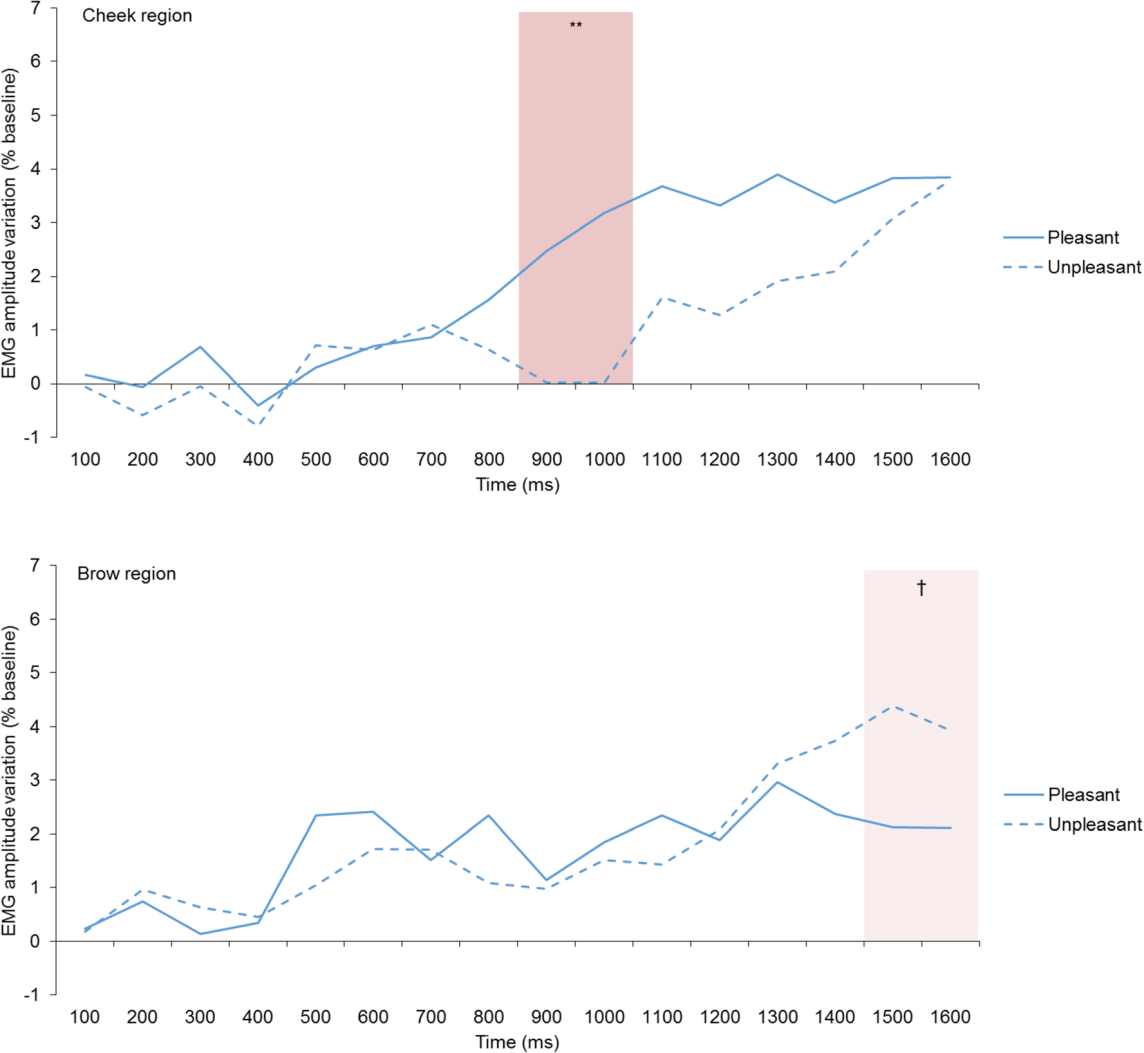
Facial EMG responses on the cheek and the brow regions elicited by the IP manipulations

Greenhouse-Geisser correction was applied whenever the assumption of sphericity was violated. Specific directed hypotheses on the timing of the main effects of IP and GC were tested one-tailed. In each case, the respective intervention has been marked in the results tables. As there were no formal predictions for interaction effects of IP × Modality and GC × Modality, two-sided testing was applied for exploration. To counteract the problem of multiple testing, we determined significance according to the Benjamini-Hochberg criterion (BH; Benjamini & Hochberg, 1995) by setting the False Discovery Rate to .05, with positive or negative direction of the effect according to the prediction. Only effects for which the *p* values were inferior to the critical level of the BH procedure are reported as significant. The uncorrected degrees of freedom, the appropriate *p* values (one- or two-sided), and the effect sizes (partial *ŋ*^2^) are reported in “Results” (Tables 2 and 3). All tests were computed in IBM SPSS Statistics 25, specifying an alpha level of 5%. While we show the data for all 16 measured time points for reference in Fig. 2, we report significance tests only for the period after 400 ms for which we have theoretical predictions (see above).

**Table 2:** Main effects for the repeated measures MANOVA for intrinsic pleasantness (IP) by modality for each facial region

		Cheek region			Brow region		
Factor (df)	Time interval (ms)	*F*	*p*	*ŋ*^2^	*F*	*p*	*ŋ*^2^
IP (1, 36)	400	0.22		0.01	0.03		0.00
	500			0.01	1.46		0.04
	600	0.01		0.00	0.57		0.02
	700	0.06		0.00	0.05		0.00
	800	1.51		0.04	0.98		0.03
	900	6.22	**	0.15	0.02		0.00
	1000	7.59	**	0.17	0.11		0.00
	1100	2.58		0.07	0.95		0.03
	1200	2.39		0.06	0.02		0.00
	1300	1.57		0.04	0.11		0.00
	1400	0.46		0.01	1.09		0.03
	1500	0.13		0.00	2.08	†	0.05
	1600	0.00		0.00	1.63	†	0.04
Modality (2, 72)	400	1.29		0.03	6.81	**	0.16
	500	0.22		0.01	7.32	**	0.17
	600	0.14		0.00	2.24		0.06
	700	1.30		0.03	1.10		0.03
	800	1.08		0.03	1.24		0.03
	900	1.31		0.04	2.24		0.06
	1000	3.71	*	0.09	0.34		0.01
	1100	3.65	*	0.09	0.11		0.00
	1200	5.41	**	0.13	1.55		0.04
	1300	6.46	**	0.15	0.18		0.00
	1400	3.91	*	0.10	0.04		0.00
	1500	4.50	*	0.11	0.07		0.00
	1600	4.51	*	0.11	0.08		0.00

Note: *N* = 37; *F* = uncorrected *F* values, *p* = Benjamini-Hochberg corrected significance levels; *ŋ*^2^ = effect sizes (partial eta squared). The complete set of results (including interaction effects) can be found in Table S2 in the SOM

†*p* < .10

*p < .05

**p < .01

**Table 3:** Main effects for the repeated measures MANOVA for goal conduciveness (GC) by modality for each facial region

Cheek region					Brow region		
Factor (*df*)	Time interval (ms)	*F*	*p*	*ŋ*^2^	*F*	*p*	*ŋ*^2^
GC (1, 36)	400	0.01		0.00	0.08		0.00
	500	0.22		0.01	0.08		0.00
	600	0.01		0.00	0.48		0.01
	700	0.92		0.03	1.37		0.04
	800	0.79		0.02	0.14		0.00
	900	0.82		0.02	0.24		0.01
	1000	2.17	†	0.06	1.20		0.03
	1100	3.22	*	0.08	1.71	†	0.05
	1200	3.77	*	0.09	1.62	†	0.04
	1300	4.63	*	0.11	1.94	†	0.05
	1400	4.51	*	0.11	2.04	†	0.05
	1500	3.24	*	0.08	1.85	†	0.05
	1600	3.35	*	0.09	2.04	†	0.05
Modality (2, 72)	400	1.18		0.03	0.55		0.01
	500	0.03		0.00	1.59		0.04
	600	0.58		0.02	3.61	*	0.09
	700	0.30		0.01	5.74	**	0.14
	800	0.13		0.00	5.55	**	0.13
	900	0.77		0.02	4.48	*	0.11
	1000	1.40		0.04	2.69		0.07
	1100	5.01	**	0.12	0.60		0.02
	1200	7.10	**	0.16	1.10		0.03
	1300	8.18	***	0.19	1.07		0.03
	1400	7.37	**	0.17	1.57		0.04
	1500	7.93	***	0.18	1.30		0.03
	1600	8.26	***	0.19	1.20		0.03

Note. *N* = 37. *F* = uncorrected *F* values, *p* = Benjamini-Hochberg corrected significance levels; *ŋ*^2^ = effect sizes (partial eta squared). The complete set of results (including interaction effects) can be found in Table S2 in the SOM

†*p* < .10

*p < .05

**p < .01

*** *p* < .001

## Results

A post-hoc power analysis using GPower (Heinrich Heine University of Düsseldorf, 2020, https://gpower.hhu.de/; Faul, Erdfelder, Lang & Buchner, 2007; Faul, Erdfelder, Buchner & Lang, 2009) was carried out for *F* tests and “MANOVA: repeated measures, within factors”. As approximation method, Pillai V and the recommended algorithm by O’Brien & Shieh (1999) were used. Based on the results of *N* = 37 participants,^3^ we chose as input parameter a partial *ŋ*^2^ = 0.15. On this basis, an effect size *f* = 0.42 was calculated. Further, our design has one group, the present analysis is based on six measurements (2 × 3 repeated measures design), and a medium correlation among the repeated measures (*r* = 0.3) was chosen. The power analysis yielded an achieved high power (1 − *β* error probability) of 0.9999574.

The results of the univariate within-subjects test for the 13 consecutive 100-ms time intervals between 400 ms and 1600 ms after stimulus onset are reported in Tables 2 and 3, separately for the IP and the GC effects, including the modality main effects for the respective condition. Given the virtual absence of interaction effects, we do not report the respective data in detail but show the results in Tables S2 and S3 as well as in Fig. S1 in the SOM. With respect to our predictions, we focus on the timing and the patterning of the appraisal main effects. Modality main effects are discussed in an exploratory fashion.

### IP Effects

#### Cheek Region

The analysis over the cheek region revealed significantly higher activity for pleasant compared with unpleasant stimuli at 900 and 1000 ms (see Table 2, column 1; Fig. 2: upper panel), in line with our predictions. The amplitude of the response (in %-change scores relative to baseline) was relatively low and the onset somewhat later than predicted.

#### Brow Region

There is little evidence for the predicted higher muscle activity in response to unpleasant stimuli relative to pleasant stimuli except for trends at 1500 ms and 1600 ms (see Table 2, column 2 and Fig. 2, lower panel). The onset occurred later than expected on the basis of the literature.

### GC Effects

**Fig. 3:**
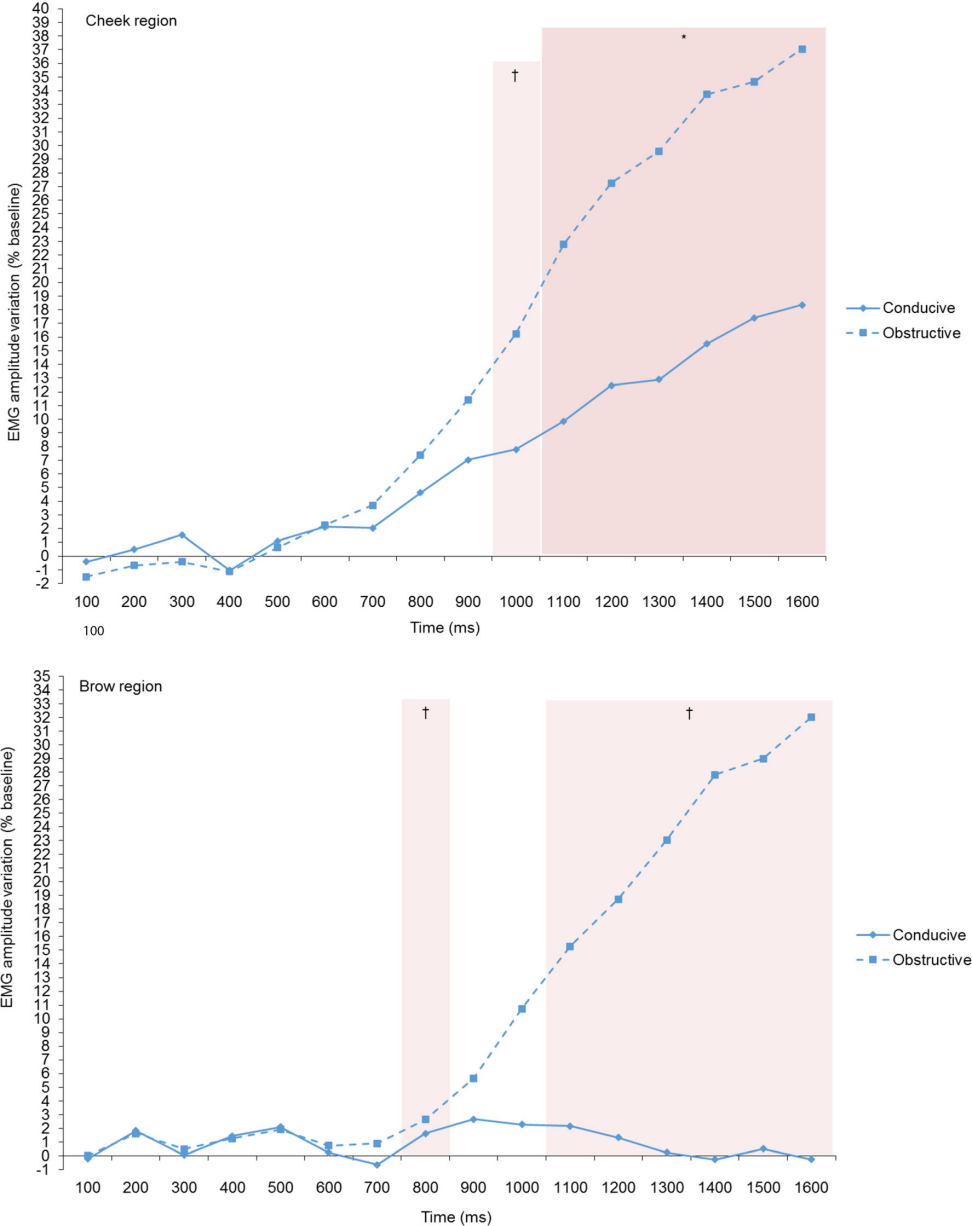
Facial EMG responses on the cheek and the brow regions elicited by the GC manipulations

Before reviewing the results in detail, note that the magnitude of the muscle activity changes (in %-change scores relative to baseline) is much higher in the GC condition than it is for the IP condition (see differences between Figs. 2 and 3, and between left and right side of Fig. S1 in the SOM). We will comment on this finding in the “Discussion”.

#### Cheek Region

At 1000 ms, there was a tendency for higher cheek region activity in the obstructive (incorrect) condition than in the conducive condition, which reached significance at all time points between 1100 ms and 1600 ms (Table 3, column 1; Fig. 3). This corresponds to the prediction in Table 1 under the assumption that rather than the zygomaticus major muscle, this measure reflects neighboring muscles around the mouth region (see the discussion in “Material and Method”).

#### Brow Region

Consistent with the CPM predictions, we found a tendency for higher brow region activity in the obstructive (“incorrect” feedback) as compared to the conducive (“correct” feedback) condition. It did not reach significance but remained at the tendency level (below *p* < .10) at all time points between 1100 and 1600 ms (Table 3, column 2; Fig. 3). This is surprising since, as shown in Fig. 3, the intensity of the activation is virtually identical with comparable values in the cheek region, for which the difference between obstructive and conducive appraisal is significant. In contrast, for the brow region, at a similar intensity and a much larger difference to the conducive condition, the difference for the obstruction condition remains only marginally significant, possibly due to greater interindividual variance.

In the hypothesis section (see above), it was mentioned that one might expect a longer duration for the effects in the GC appraisal outcomes, as the cognitive processes involved might be more complex. This is indeed the case as shown in Figs. 2 and 3.

### Sensory Modality Effects

#### Modality Main Effects

### IP Appraisal

There are some highly significant modality effects for the IP manipulation in the range between 1000 ms and 1600 ms for the cheek region (see Table 2, column 1). These effects are mainly due to generally higher muscle activity changes in the olfactory condition (see Table S3 and Fig. S1 in the SOM).

### GC Appraisal

Over the cheek region, we find strongly significant modality main effects between 1200 ms and 1600 ms (see Table 3, as well as Table S3 and Fig. S1 in the SOM), mostly due to face and voice stimuli producing stronger effects of muscle activity changes than odor stimuli. Over the brow region, we find significant differences between 600 and 900 ms (see Table 3 second column; and Table S3 and Fig. S1 in the SOM) with the visual condition producing stronger activation than the voice or the odor stimuli. The origin of these unpredicted effects is difficult to ascertain, given that the GC manipulation was independent of the modality. One possible explanation might be that participants had stronger expectations regarding their chances to make a correct judgment, as it may be more common to attempt to infer personality from facial (and to some extent, vocal) features as compared to basing the judgment on presumably preferred odors. Strong positive expectations are likely to produce a more intense appraisal of goal obstruction, and consequently stronger responses in the facial musculature.

#### Interaction Effects of Modality × IP and Modality × GC

With one exception, none of these interaction effects reached significance. In the interest of conciseness, we decided not to document the detailed results in this report (but see Table S2, Tables S3, and Fig. S1 in the SOM).

## Discussion

As outlined in “Introduction,” splitting up the unitary macro-valence factor that dominates much of the emotion literature into several micro-valences, especially IP and GC, is of major importance for emotion research. Among other things, it allows a more principled approach to study the frequent occurrence of mixed or blended emotions. In terms of underlying mechanisms, we believe that this phenomenon is best explained by separate effects of micro-valences in the appraisal process. Our study aims to add further knowledge to the architecture of micro-valence appraisal with respect to their effect on the timing and the patterning on facial muscle changes. The CPM offers an architecture for such a process and concrete predictions on this micro-valence sequence are proposed. While these CPM predictions have been generally strongly supported in previous studies, the latter have used different designs and measures in different modalities. Here, we report the first study that has attempted to test the distinctiveness and the sequence in which IP and GC are processed in a single study, with the same group of participants, exploring for the first time comparable operationalizations of IP and GC across different modalities.

In this study, we focused on whether the two types of micro-valences—IP and GC—(e.g., Scherer, 2009; Shuman et al., 2013) can be differentiated in facial EMG recordings irrespective of the modality of stimulus presentation. Embedded in a social judgment task, stimuli were presented in three sensual modalities: vision, audition, and olfaction. We predicted that appraisal results of IP, as compared to GC, would show (a) an earlier onset (based on the CPM sequence hypothesis) and (b) a subtly different response pattern for facial muscle responses (based on CPM componential patterning predictions). In addition, potential effects of the presentation modality of the stimuli were explored.

As to the central aims listed in the “Introduction,” we draw the following three major conclusions: (1) the results support the prediction of the CPM that IP and GC effects can be clearly differentiated regarding their timing and response patterning; (2) while the response patterns overlap, as found in earlier work, our results suggest that for IP, pleasantness outcome has a stronger impact on the cheek region (most probably, smiling produced by the zygomaticus major muscle) than on the brow region. For GC, obstruction appraisal drives both the activity of the brow region (probably frowning produced by the corrugator supercilii muscle) and the cheek region (probably upper lip raising by the levator labii superiori muscle plus tightening and pressing of the lips by the orbicularis oris muscle). A conduciveness result for GC appraisal seems to affect the facial muscles less strongly than an obstructive result. The predicted sequence of IP preceding GC is again clearly confirmed. As to the exact timing, our results suggest that it is advisable to assume larger time windows than what is suggested by EEG results, for example, IP responses between 400 and 1000 ms and GC responses between 1000 and 1600 ms. (3) As to modality effects, odor stimuli seem to produce stronger facial muscle activation than faces and voice samples. As we did not find significant interaction effects between modality and IP and modality and GC, the results on IP/GC differences seem to have a high degree of generalizability. However, some of the strong modality main effects we observed suggest that modality (e.g., especially unpleasant odors and portraits) may have an influence on the intensity of the responses.

We cannot exclude that some of the modality results might be due to the task design. The cover story may have modified the importance of the manipulated stimuli for IP appraisal. Given that participants performed a social judgment task, it is possible that the feedback stimuli were of higher (task) relevance than the faces, voices, and odors, especially as winning was involved. The finding that the extent of muscle activity changes was systematically higher in the correct/incorrect feedback conditions seems to support this assumption. In each situation—as a function of a person’s current goals and needs—the stimuli seem to be categorized for their importance through an implicit weighting process. In our study, participants might have focused on features of the stimuli that would help them to judge correctly whether the person (for whom they have seen the face, heard the voice, or smelled their preferred odor) is introverted or extraverted. This attentional shift might have prioritized the processing of task-relevant information (e.g., performance feedback) instead of the hedonic information. It is also possible that the experimental task could have initiated a reevaluation process (cumulative appraisal process) due to motivational weighting of the relevant cues. Therefore, it seems that a relevance appraisal check is always performed and is inherent to the task that one focusses on (e.g., Moors, Houwer, Hermans & Eelen, 2005; Schacht, Werheid & Sommer, 2008).

The results for modulating effects of the perception channel in which the stimuli were perceived suggest that unpleasant odors initiate the strongest muscle responses over the cheek region. From an evolutionary and neuroanatomical perspective, the olfactory system is directly involved in detecting unpleasant stimuli that could indicate harm. Despite our efforts to equalize the arousing impact of our stimuli across modalities, the olfactory stimuli may have elicited—due to anatomical specificities—stronger muscle activity changes.

### Limitations

The operationalization of IP via the relative pleasantness of faces and voices (to create a comparable interpersonal context for these conditions) may be problematic. Despite our careful pretesting to create a homogenous set of the stimuli regarding their rated pleasantness and arousal, there may well be strong interindividual differences affecting this appraisal check. For example, the greater biological and sociosexual significance of faces may trigger more complex appraisals (see Gerger, Leder, Tinio & Schacht, 2011).

Another obvious limitation is the relative lack of specificity concerning the muscles involved due to imprecision of the surface electrode measurement by facial region. However, as the muscle innervations detected with this method are generally not visible in the face, for the moment, there is no alternative, if one wants to measure the important facial responses triggered by micro-valence appraisals. Obviously, as always, further work is needed to disentangle these potential influencing factors and to improve on the experimental design and the EMG measurement.

### Future Research

These results raise several important issues for future research. While there can be little doubt about the distinctiveness and sequential occurrence of IP and GC, there is a need for further research on the exact nature of the response patterning in the facial musculature. As in earlier work, we note that the differences with respect to the muscle regions are relatively subtle. This is not surprising as in many cases the same face regions and sometimes, the same muscles are involved, as suggested in the predictions. Nevertheless, our design allowed to directly compare the relative effect of distinct IP and GC outcomes on different muscle regions. This raises the question of the relative strength of the impact of the appraisal results on the facial musculature as illustrated in our results. One important factor is certainly the relative importance and urgency of the action tendencies activated by IP and GC. If there is an immediate danger indicated by a bad odor, a visual flash, or a loud scream, the IP appraisal should prime the responses in order to activate immediate avoidance responses (one of the reasons for the temporal primacy of the IP appraisal). This may depend on the intensity of the stimulation. If it is low, it may be more important to first check on the goal conduciveness of the situation to activate an appropriate action tendency. If it is high, the best strategy might be to avoid the danger at all cost. Further work should focus on issues of the relative importance and functionality (as assessed by the relevance check in the appraisal sequence) and the urgency of specific action tendencies.

Clearly, our results are strongly affected by the nature of the manipulations and the specific context created. Our findings invite important theoretical speculations for further research. For example, when people have a clear goal as in our GC condition, they are likely to be particularly sensitive to information that is directly relevant to that goal (and perhaps tend to ignore other information), as suggested by the larger effects in the GC condition. IP effects may be more important in situations where people do not have an overriding goal or motivational urge. In addition, they may be greatest when participants do not have a task to distract them from processing the sensory information. In our case, in the IP condition, participants still had to judge whether the stimulus reflected introversion or extraversion of the respective person (which requires concentration and non-negligible cognitive inference). This factor could be responsible for the relatively feeble effect of the IP differences.

Thus, it would be useful for future research to examine more systematically other possible reasons next to the eliciting impact of micro-valence appraisal for differential onsets and durations of facial muscle movement in response to IP and GC. It is to be expected that, in general, the appraisal of intrinsic qualities will be faster and shorter. This is probably linked to the complexity of the factors to be considered in appraisal and on the level of processing involved (see Leventhal & Scherer, 1987). Moreover, it would also be promising to manipulate other important appraisal dimensions in this context, for example, novelty (for IP) and expectancy (for GC). Other important micro-valences to systematically examine are relevance and coping ability. Such an extension of manipulated micro-valences would be important to gain more insight regarding their mediating effects on the onset, the duration, and the (facial) response patterning.

Another important aspect of future research agendas should be further development of the surface facial EMG methodology to allow more reliable measurement of different muscles or muscle groups in the brow and cheek regions, including the possibility of recording the EMG outside of the laboratory. While there are some promising reports (e.g., Inzelberg, Rand, Steinberg, David-Pur & Hanein, 2018), further development is urgently needed.

## Conclusion

The results show clearly distinct facial EMG responses and timing patterns for both types of micro-valence, confirming the prediction that they are independent, consecutive appraisal processes. Moreover, the lack of interaction effects with the sensory stimulus modality suggests high generalizability of the underlying appraisal mechanisms across different perception channels.

Overall, the findings correspond to the title of an earlier study by our group: the effects of IP and GC are “somewhat similar, but not identical” (Aue & Scherer, 2011). Most importantly, we replicate the major indicator of a difference between these two micro-valences which is their timing. We find the predicted sequential effects of the onset times of IP and GC, but these were only significant over the cheek region (IP effects starting at 900 ms after stimulus onset, GC effects at 1.1 s). Over the brow region, only tendencies were obtained, and the effects were less strong. While the IP effects occur somewhat later than reported in earlier studies (e.g., Achaibou, Pourtois, Schwartz & Vuilleumier, 2008; Delplanque et al., 2009), they preceded GC effects for about 200 to 300 ms (corresponding to previous EEG findings; Gentsch et al., 2013). Furthermore, as expected, IP effects are also shorter than GC effects, corresponding to the lower level and the differential complexity of the cognitive processes involved (see Leventhal & Scherer, 1987). As to the response patterns of the different facial muscles, overall, there was a tendency for pleasant stimuli to activate the cheek region and for goal obstructive stimuli (which had a much stronger effect than conducive stimuli) to affect both the brow and the cheek regions. The results are largely in line with the theoretical predictions shown in Table 1 and generally replicate earlier findings.

The important addition in this work is the investigation of modality effects of the micro-valence appraisal manipulations. While there is a strong main effect for the odor modality producing stronger cheek region muscle activity changes in both the IP and GC conditions, virtually, none of the interaction effects between the type of micro-valence and the modality reached significance. The pronounced effect of unpleasant odors in contrast to unpleasant faces and voices probably reflects the powerful effects of olfaction on emotional experience often referred to in the literature (e.g., Ehrlichman & Bastone, 1992). Nonetheless, the response patterning was largely independent from the perception channel in which appraisal information were assessed. This suggests that research findings manipulating appraisal in different modalities and experimental designs can be generalized.

Further reports on the remaining response channels measured in this work are in preparation and will eventually allow to also examine other central predictions of the CPM such as the degree of synchronization among emotion components whose responses are postulated to be driven by appraisal checks (e.g., Scherer, 2001, 2009). It is to be hoped that further research in this area will adopt, more readily than in the past, complex process-oriented designs that allow a more comprehensive experimental examination of the appraisal-driven emotion process.

## Electronic Supplementary Material

ESM 1 (DOCX 203 kb)

## Footnotes

1 We used questionnaires that measure hedonic traits (French version of the Snaith-Hamilton Pleasure Scale, Gaillard, Gourion & Llorca, 2013), behavioral traits (French version of the behavioral inhibition, behavioral activation scale, Caci, Deschaux & Baylé, 2007; Carver & White, 1994), personality traits (French version of the Big Five Inventory 10 items, Plaisant, Courtois, Réveillère, Mendelsohn & John, 2010; Rammstedt & John, 2007), and emotion regulation (French version of the Emotion Regulation Questionnaire, Christophe, Antoine, Leroy & Delelis, 2009; Gross & John, 2003).

2 In this study, several dependent psychophysiological measures were recorded. Here, we present only the EMG data. The report of the results of the other measures is in preparation.

3 In total, 48 participants took part in the present study. Before processing the EMG data, 11 participants were excluded (four participants were too sleepy, one data set of one participant had too much noise coming from construction work from the neighboring laboratory room, one participant turned out to be ambidextrous, one participant came only to one session, and the data of four participants were excluded because of too much noise in the recorded EMG data due to technical issues). Thus, *N* = 37 remained for the statistical analyses.

## Additional Information

### Data Availability

The data from this study can be found at https://osf.io/tegb9/.
