# Supplementary material for: Temporal Unfolding of Micro-valences in Facial Expression Evoked by Visual, Auditory, and Olfactory Stimuli
Source: Affect Sci. 2020 Nov 13;1(4):208–24. doi: 10.1007/s42761-020-00020-y (PMC7717056; doi:10.1007/s42761-020-00020-y)
Supplement: Supplementary file 1 — (DOCX 203 kb) [file 42761_2020_20_MOESM1_ESM.docx]

Supplemental Online Material (SOM) for

Temporal unfolding of micro-valences in facial expression evoked by visual, auditory, and olfactory stimuli

Kornelia Gentsch, Ursula Beermann, Lingdan Wu, Stéphanie Trznadel, and Klaus R. Scherer

Table S1

Rating of felt pleasantness for all experimental stimuli

| Modality | *M (SD)* pleasant stimuli | *M (SD)*  unpleasant stimuli | *df* | *t* | *p* | 95% CI | | |
| --- | --- | --- | --- | --- | --- | --- | --- | --- |
|  |  |  |  |  |  | Lower | Upper | Cohen’s *d* |
| Vision | 6.17 (0.69) | 3.65 (0.93) | 36 | 11.53 | .000 | 2.08 | 2.97 | 1.90 |
| Audition | 5.71 (0.71) | 3.43 (0.91) | 36 | 10.91 | .000 | 1.92 | 2.80 | 1.79 |
| Olfaction | 5.98 (0.91) | 3.95 (0.72) | 36 | 11.77 | .000 | 1.71 | 2.42 | 1.94 |

*Note*. *N* = 37. As a manipulation check, at the end of the experiment, and only after the second session, participants rated each stimulus concerning the felt pleasantness (nine-point scale from 1 (very unpleasant) to 9 (very pleasant).

Table S2

Main and interaction effects of the repeated measures MANOVAs

| **CHEEK REGION** | | | | | | | | | | | | | | | | | | | | | | | | | | | | | | | | | | | | | | | | | | | | | | | | | | | | | | | |  |  |  |  |
| --- | --- | --- | --- | --- | --- | --- | --- | --- | --- | --- | --- | --- | --- | --- | --- | --- | --- | --- | --- | --- | --- | --- | --- | --- | --- | --- | --- | --- | --- | --- | --- | --- | --- | --- | --- | --- | --- | --- | --- | --- | --- | --- | --- | --- | --- | --- | --- | --- | --- | --- | --- | --- | --- | --- | --- | --- | --- | --- | --- |
| ***IP manipulation*** | |  |  | | |  | | |  | | |  |  | | | |  | | | |  | | | |  | | | |  | | | |  | | | |  | | | | |  | | | | |  | | |  | | | | | |  |  |  |  |
| Modality main effects | | |  | | |  | | |  | | | IP main effects | | | | |  | | | |  | | | |  | | | |  | | | | Modality × IP interaction effects | | | | | | | | | | | | | | | | |  | | | | | |  |  |  |  |
| Time (ms) | *F* | *p* |  | | ŋ² | | |  | | | Time (ms) | | | | *F* | | | | *p* | | | |  | | | | eta | | | |  | | | | Time (ms) | | | | | *F* | | | | | *p* | | | | |  | | | | ŋ² | | | | |  |
| 400 | 1.29 | .28 |  | | 0.03 | | |  | | | 400 | | | | 0.22 | | | | .32 | | | |  | | | | 0.01 | | | |  | | | | 400 | | | | | 0.78 | | | | | .46 | | | | |  | | | | 0.02 | | | | |  |
| 500 | 0.22 | .80 |  | | 0.01 | | |  | | | 500 | | | | 0.29 | | | | .30 | | | |  | | | | 0.01 | | | |  | | | | 500 | | | | | 2.81 | | | | | .07 | | | | |  | | | | 0.07 | | | | |  |
| 600 | 0.14 | .87 |  | | 0.00 | | |  | | | 600 | | | | 0.01 | | | | .47 | | | |  | | | | 0.00 | | | |  | | | | 600 | | | | | 0.04 | | | | | .97 | | | | |  | | | | 0.00 | | | | |  |
| 700 | 1.30 | .28 |  | | 0.03 | | |  | | | 700 | | | | 0.06 | | | | .40 | | | |  | | | | 0.00 | | | |  | | | | 700 | | | | | 1.11 | | | | | .33 | | | | |  | | | | 0.03 | | | | |  |
| 800 | 1.08 | .34 |  | | 0.03 | | |  | | | 800 | | | | 1.51 | | | | .11 | | | |  | | | | 0.04 | | | |  | | | | 800 | | | | | 0.42 | | | | | .66 | | | | |  | | | | 0.01 | | | | |  |
| 900 | 1.31 | .27 |  | | 0.04 | | |  | | | 900 | | | | 6.22 | | | | .01 | | | | ** | | | | 0.15 | | | |  | | | | 900 | | | | | 0.43 | | | | | .65 | | | | |  | | | | 0.01 | | | | |  |
| 1000 | 3.71 | .03 | * | | 0.09 | | |  | | | 1000 | | | | 7.59 | | | | .01 | | | | ** | | | | 0.17 | | | |  | | | | 1000 | | | | | 1.45 | | | | | .24 | | | | |  | | | | 0.04 | | | | |  |
| 1100 | 3.65 | .03 | * | | 0.09 | | |  | | | 1100 | | | | 2.58 | | | | .18 | | | |  | | | | 0.07 | | | |  | | | | 1100 | | | | | 1.17 | | | | | .32 | | | | |  | | | | 0.03 | | | | |  |
| 1200 | 5.41 | .01 | ** | | 0.13 | | |  | | | 1200 | | | | 2.39 | | | | .13 | | | |  | | | | 0.06 | | | |  | | | | 1200 | | | | | 2.24 | | | | | .11 | | | | |  | | | | 0.06 | | | | |  |
| 1300 | 6.46 | .01 | ** | | 0.15 | | |  | | | 1300 | | | | 1.57 | | | | .22 | | | |  | | | | 0.04 | | | |  | | | | 1300 | | | | | 0.24 | | | | | .79 | | | | |  | | | | 0.01 | | | | |  |
| 1400 | 3.91 | .04 | * | | 0.10 | | |  | | | 1400 | | | | 0.46 | | | | .50 | | | |  | | | | 0.01 | | | |  | | | | 1400 | | | | | 0.94 | | | | | .39 | | | | |  | | | | 0.03 | | | | |  |
| 1500 | 4.50 | .03 | * | | 0.11 | | |  | | | 1500 | | | | 0.13 | | | | .72 | | | |  | | | | 0.00 | | | |  | | | | 1500 | | | | | 0.75 | | | | | .48 | | | | |  | | | | 0.02 | | | | |  |
| 1600 | 4.51 | .03 | * | | 0.11 | | |  | | | 1600 | | | | 0.00 | | | | .98 | | | |  | | | | 0.00 | | | |  | | | | 1600 | | | | | 0.97 | | | | | .39 | | | | |  | | | | 0.03 | | | | |  |
|  |  |  |  | | |  | | |  | | |  |  | | | |  | | | |  | | | |  | | | |  | | | |  | | | |  | | | | |  | | | | |  | | |  | | | | | |  |  |  |  |
| ***GC manipulation*** | | |  | | |  | | |  | | |  |  | | | |  | | | |  | | | |  | | | |  | | | |  | | | |  | | | | |  | | | | |  | | |  | | | | | |  |  |  |  |
| Modality main effects | | |  | | |  | | |  | | | GC main effects | | | | |  | | | |  | | | |  | | | |  | | | | Modality × GC interaction effects | | | | | | | | | | | | | | | | | | | | | | |  |  |  |  |
| Time (ms) | *F* | *p* |  | ŋ² | | |  | | | Time (ms) | | | | *F* | | | | *p* | | | |  | | | | ŋ² | | | |  | | | | Time (ms) | | | | | *F* | | | | | *p* | | | | |  | | | | ŋ² | | | | |  |  |
| 400 | 1.18 | .31 |  | 0.03 | | |  | | | 400 | | | | 0.01 | | | | .92 | | | |  | | | | 0.00 | | | |  | | | | 400 | | | | | 0.05 | | | | | .95 | | | | |  | | | | 0.00 | | | | |  |  |
| 500 | 0.03 | .97 |  | 0.00 | | |  | | | 500 | | | | 0.22 | | | | .64 | | | |  | | | | 0.01 | | | |  | | | | 500 | | | | | 0.46 | | | | | .64 | | | | |  | | | | 0.01 | | | | |  |  |
| 600 | 0.58 | .56 |  | 0.02 | | |  | | | 600 | | | | 0.01 | | | | .91 | | | |  | | | | 0.00 | | | |  | | | | 600 | | | | | 0.40 | | | | | .67 | | | | |  | | | | 0.01 | | | | |  |  |
| 700 | 0.30 | .74 |  | 0.01 | | |  | | | 700 | | | | 0.92 | | | | .34 | | | |  | | | | 0.03 | | | |  | | | | 700 | | | | | 0.70 | | | | | .50 | | | | |  | | | | 0.02 | | | | |  |  |
| 800 | 0.13 | .88 |  | 0.00 | | |  | | | 800 | | | | 0.79 | | | | .38 | | | |  | | | | 0.02 | | | |  | | | | 800 | | | | | 0.89 | | | | | .41 | | | | |  | | | | 0.02 | | | | |  |  |
| 900 | 0.77 | .47 |  | 0.02 | | |  | | | 900 | | | | 0.82 | | | | .37 | | | |  | | | | 0.02 | | | |  | | | | 900 | | | | | 0.83 | | | | | .44 | | | | |  | | | | 0.02 | | | | |  |  |
| 1000 | 1.40 | .25 |  | 0.04 | | |  | | | 1000 | | | | 2.17 | | | | .08 | | | | † | | | | 0.06 | | | |  | | | | 1000 | | | | | 1.84 | | | | | .17 | | | | |  | | | | 0.05 | | | | |  |  |
| 1100 | 5.01 | .01 | ** | 0.12 | | |  | | | 1100 | | | | 3.22 | | | | .04 | | | | * | | | | 0.08 | | | |  | | | | 1100 | | | | | 2.47 | | | | | .09 | | | | |  | | | | 0.06 | | | | |  |  |
| 1200 | 7.10 | .01 | ** | 0.16 | | |  | | | 1200 | | | | 3.77 | | | | .03 | | | | * | | | | 0.09 | | | |  | | | | 1200 | | | | | 2.46 | | | | | .09 | | | | |  | | | | 0.06 | | | | |  |  |
| 1300 | 8.18 | .00 | *** | 0.19 | | |  | | | 1300 | | | | 4.63 | | | | .02 | | | | * | | | | 0.11 | | | |  | | | | 1300 | | | | | 3.24 | | | | | .05 | | | | | * | | | | 0.08 | | | | |  |  |
| 1400 | 7.37 | .01 | ** | 0.17 | | |  | | | 1400 | | | | 4.51 | | | | .02 | | | | * | | | | 0.11 | | | |  | | | | 1400 | | | | | 1.58 | | | | | .21 | | | | |  | | | | 0.04 | | | | |  |  |
| 1500 | 7.93 | .01 | *** | 0.18 | | |  | | | 1500 | | | | 3.24 | | | | .04 | | | | * | | | | 0.08 | | | |  | | | | 1500 | | | | | 1.05 | | | | | .36 | | | | |  | | | | 0.03 | | | | |  |  |
| 1600 | 8.26 | .00 | *** | 0.19 | | |  | | | 1600 | | | | 3.35 | | | | .04 | | | | * | | | | 0.09 | | | |  | | | | 1600 | | | | | 1.62 | | | | | .21 | | | | |  | | | | 0.04 | | | | |  |  |
|  |  |  |  | | |  | | |  | | |  |  | | | |  | | | |  | | | |  | | | |  | | | |  | | | |  | | | | |  | | | | |  | | |  | | | | | |  |  |  |  |
| **BROW REGION** | | | | | | | | | | | | | | | | | | | | | | | | | | | | | | | | | | | | | | | | | | | | | | | | | | | | | | | |  |  |  |  |
| ***IP manipulation*** | |  |  | | |  | | |  | | |  |  | | | |  | | | |  | | | |  | | | |  | | | |  | | | |  | | | | |  | | | | |  | | |  | | | | | |  |  |  |  |
| Modality main effects | | | | | | | | | | | | IP main effects | | | | | | | | | | | | | | | | |  | | | | Modality × IP interaction effects | | | | | | | | | | | | | | | | |  | | | | | |  |  |  |  |
| Time (ms) | *F* | *p* |  | | | ŋ² | | |  | | | Time (ms) | | | | *F* | | | | *p* | | | |  | | | | ŋ² | | | |  | | | | Time (ms) | | | | | *F* | | | | | *p* | | | | |  | | | | ŋ² | | | | |
| 400 | 6.81 | .00 | ** | | | 0.16 | | |  | | | 400 | | | | 0.03 | | | | .87 | | | |  | | | | 0.00 | | | |  | | | | 400 | | | | | 0.19 | | | | | .83 | | | | |  | | | | 0.01 | | | | |
| 500 | 7.32 | .00 | ** | | | 0.17 | | |  | | | 500 | | | | 1.46 | | | | .23 | | | |  | | | | 0.04 | | | |  | | | | 500 | | | | | 1.00 | | | | | .37 | | | | |  | | | | 0.03 | | | | |
| 600 | 2.24 | .11 |  | | | 0.06 | | |  | | | 600 | | | | 0.57 | | | | .46 | | | |  | | | | 0.02 | | | |  | | | | 600 | | | | | 0.04 | | | | | .97 | | | | |  | | | | 0.00 | | | | |
| 700 | 1.10 | .34 |  | | | 0.03 | | |  | | | 700 | | | | 0.05 | | | | .82 | | | |  | | | | 0.00 | | | |  | | | | 700 | | | | | 0.19 | | | | | .82 | | | | |  | | | | 0.01 | | | | |
| 800 | 1.24 | .29 |  | | | 0.03 | | |  | | | 800 | | | | 0.98 | | | | .33 | | | |  | | | | 0.03 | | | |  | | | | 800 | | | | | 0.80 | | | | | .45 | | | | |  | | | | 0.02 | | | | |
| 900 | 2.24 | .11 |  | | | 0.06 | | |  | | | 900 | | | | 0.02 | | | | .89 | | | |  | | | | 0.00 | | | |  | | | | 900 | | | | | 2.09 | | | | | .13 | | | | |  | | | | 0.05 | | | | |
| 1000 | 0.34 | .72 |  | | | 0.01 | | |  | | | 1000 | | | | 0.11 | | | | .37 | | | |  | | | | 0.00 | | | |  | | | | 1000 | | | | | 0.39 | | | | | .68 | | | | |  | | | | 0.01 | | | | |
| 1100 | 0.11 | .90 |  | | | 0.00 | | |  | | | 1100 | | | | 0.95 | | | | .17 | | | |  | | | | 0.03 | | | |  | | | | 1100 | | | | | 0.72 | | | | | .49 | | | | |  | | | | 0.02 | | | | |
| 1200 | 1.55 | .22 |  | | | 0.04 | | |  | | | 1200 | | | | 0.02 | | | | .44 | | | |  | | | | 0.00 | | | |  | | | | 1200 | | | | | 1.84 | | | | | .17 | | | | |  | | | | 0.05 | | | | |
| 1300 | 0.18 | .84 |  | | | 0.00 | | |  | | | 1300 | | | | 0.11 | | | | .37 | | | |  | | | | 0.00 | | | |  | | | | 1300 | | | | | 1.09 | | | | | .34 | | | | |  | | | | 0.03 | | | | |
| 1400 | 0.04 | .97 |  | | | 0.00 | | |  | | | 1400 | | | | 1.09 | | | | .15 | | | |  | | | | 0.03 | | | |  | | | | 1400 | | | | | 1.80 | | | | | .17 | | | | |  | | | | 0.05 | | | | |
| 1500 | 0.07 | .93 |  | | | 0.00 | | |  | | | 1500 | | | | 2.08 | | | | .08 | | | | † | | | | 0.05 | | | |  | | | | 1500 | | | | | 2.92 | | | | | .06 | | | | | † | | | | 0.07 | | | | |
| 1600 | 0.08 | .92 |  | | | 0.00 | | |  | | | 1600 | | | | 1.63 | | | | .11 | | | | † | | | | 0.04 | | | |  | | | | 1600 | | | | | 2.60 | | | | | .08 | | | | | † | | | | 0.07 | | | | |
|  |  |  |  | | |  | | |  | | |  | | | |  | | | |  | | | |  | | | |  | | | |  | | | |  | |  | | | | |  | | | | |  | | | |  | | | | |  |  |  |
| ***GC manipulation*** | | | | | | | | | | | | | | | | | | | | | | | | | | | | | | | | | | | | | | | | | | | | | | | | | | | | | | | |  |  |  |  |
| Modality main effects | | |  | | |  | | |  | | | GC main effects | | | | |  | | | |  | | | |  | | | |  | | | | Modality × GC interaction effects | | | | | | | | | | | | | | | | | | | | | | |  |  |  |  |
| Time (ms) | *F* | *p* |  | | | ŋ² | | |  | | | Time (ms) | | | | *F* | | | | *p* | | | |  | | | | ŋ² | | | |  | | | | Time (ms) | | | | | *F* | | | | | *p* | | | | |  | | | | ŋ² | | | | |
| 400 | 0.55 | .58 |  | | | 0.01 | | |  | | | 400 | | | | 0.08 | | | | .78 | | | |  | | | | 0.00 | | | |  | | | | 400 | | | | | 0.42 | | | | | .66 | | | | |  | | | | 0.01 | | | | |
| 500 | 1.59 | .21 |  | | | 0.04 | | |  | | | 500 | | | | 0.08 | | | | .77 | | | |  | | | | 0.00 | | | |  | | | | 500 | | | | | 0.48 | | | | | .62 | | | | |  | | | | 0.01 | | | | |
| 600 | 3.61 | .04 | * | | | 0.09 | | |  | | | 600 | | | | 0.48 | | | | .49 | | | |  | | | | 0.01 | | | |  | | | | 600 | | | | | 0.45 | | | | | .64 | | | | |  | | | | 0.01 | | | | |
| 700 | 5.74 | .00 | ** | | | 0.14 | | |  | | | 700 | | | | 1.37 | | | | .25 | | | |  | | | | 0.04 | | | |  | | | | 700 | | | | | 0.40 | | | | | .67 | | | | |  | | | | 0.01 | | | | |
| 800 | 5.55 | .01 | ** | | | 0.13 | | |  | | | 800 | | | | 0.14 | | | | .71 | | | |  | | | | 0.00 | | | |  | | | | 800 | | | | | 1.20 | | | | | .31 | | | | |  | | | | 0.03 | | | | |
| 900 | 4.48 | .04 | * | | | 0.11 | | |  | | | 900 | | | | 0.24 | | | | .63 | | | |  | | | | 0.01 | | | |  | | | | 900 | | | | | 2.57 | | | | | .08 | | | | | † | | | | 0.07 | | | | |
| 1000 | 2.69 | .10 | † | | | 0.07 | | |  | | | 1000 | | | | 1.20 | | | | .14 | | | |  | | | | 0.03 | | | |  | | | | 1000 | | | | | 1.91 | | | | | .16 | | | | |  | | | | 0.05 | | | | |
| 1100 | 0.60 | .55 |  | | | 0.02 | | |  | | | 1100 | | | | 1.71 | | | | .10 | | | | † | | | | 0.05 | | | |  | | | | 1100 | | | | | 1.26 | | | | | .29 | | | | |  | | | | 0.03 | | | | |
| 1200 | 1.10 | .34 |  | | | 0.03 | | |  | | | 1200 | | | | 1.62 | | | | .11 | | | | † | | | | 0.04 | | | |  | | | | 1200 | | | | | 1.35 | | | | | .27 | | | | |  | | | | 0.04 | | | | |
| 1300 | 1.07 | .35 |  | | | 0.03 | | |  | | | 1300 | | | | 1.94 | | | | .09 | | | | † | | | | 0.05 | | | |  | | | | 1300 | | | | | 1.18 | | | | | .31 | | | | |  | | | | 0.03 | | | | |
| 1400 | 1.57 | .21 |  | | | 0.04 | | |  | | | 1400 | | | | 2.04 | | | | .08 | | | | † | | | | 0.05 | | | |  | | | | 1400 | | | | | 1.36 | | | | | .26 | | | | |  | | | | 0.04 | | | | |
| 1500 | 1.30 | .28 |  | | | 0.03 | | |  | | | 1500 | | | | 1.85 | | | | .09 | | | | † | | | | 0.05 | | | |  | | | | 1500 | | | | | 1.76 | | | | | .18 | | | | |  | | | | 0.05 | | | | |
| 1600 | 1.20 | .31 |  | | | 0.03 | | |  | | | 1600 | | | | 2.04 | | | | .08 | | | | † | | | | 0.05 | | | |  | | | | 1600 | | | | | 1.45 | | | | | .24 | | | | |  | | | | 0.04 | | | | |
|  |  |  |  | | |  | | |  | | |  | | | |  | | | |  | | | |  | | | |  | | | |  | | | |  | | | | |  | | | | |  | | | | |  | | | |  | | | | |
| *Note*: *N* = 37; *F* = uncorrected *F* values, *p* = Benjamini-Hochberg corrected significance levels, †*p* < .10, **p* < .05, ***p* < .01;  ŋ² = effect sizes (partial eta squared). | | | | | | | | | | | | | | | | | | | | | | | | | | | | | | | | | | | | | | | | | | | | | | | | | | | | | | | | | | | |
| Greenhouse-Geiser correction | | | | | | | | |  | | |  | | | |  | | | | one-sided testing | | | | | | | | | | | | | | | | | | | | |  | | | | |  | | | | |  | | | |  | | | | |

Table S3

EMG activity - Mean % change compared to baseline

Figure S1

Facial EMG responses over time on the cheek and the brow regions elicited by the IP and the GC manipulations in three different modalities


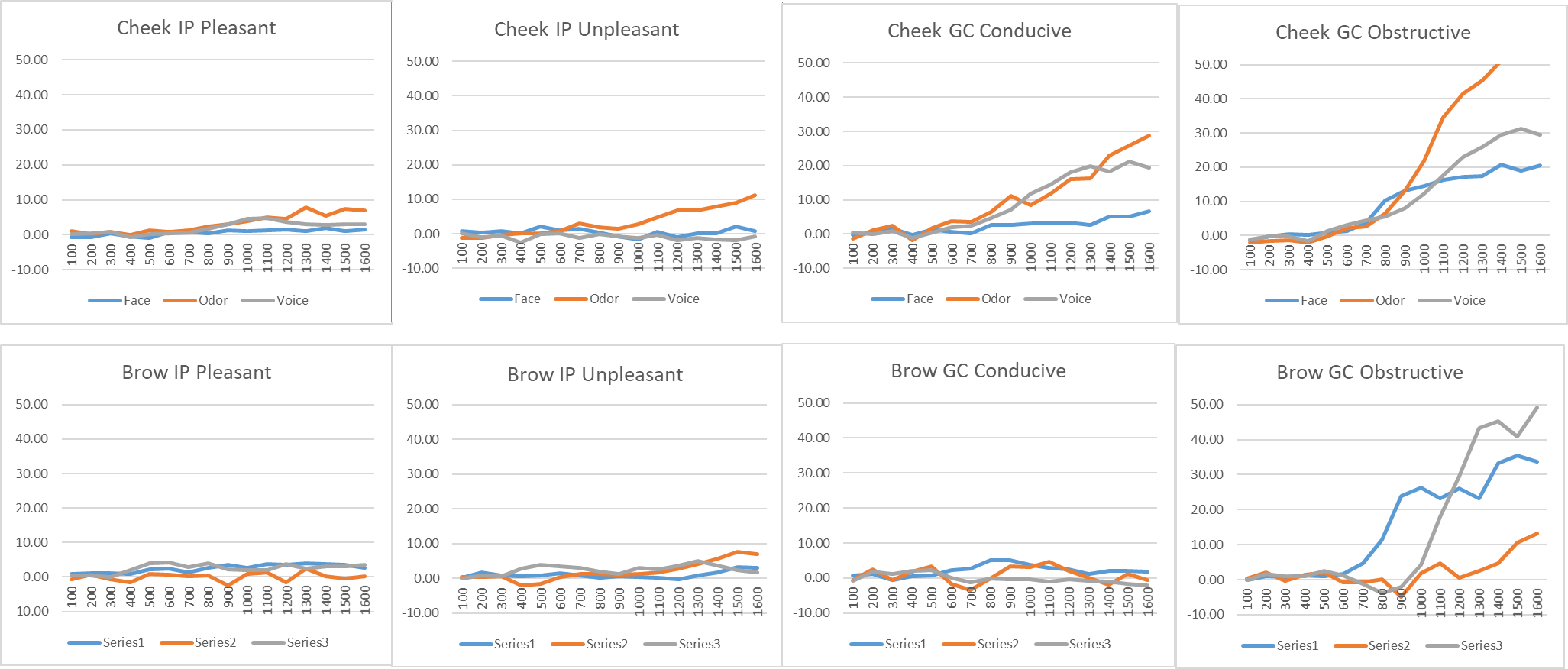


*Note*. The data of the IP (pleasant vs. unpleasant) and the GC (conducive vs. obstructive) manipulations are presented in Table S2. The modalities were: vision with face stimuli, audition with voice samples, and olfaction with odor samples.
